# Supplementary material for: OpenMM 7: Rapid development of high performance algorithms for molecular dynamics
Source: PLoS Comput Biol. 2017 Jul 26;13(7):e1005659. doi: 10.1371/journal.pcbi.1005659 (PMC5549999; doi:10.1371/journal.pcbi.1005659)
Supplement: S1 Source Code — (ZIP) [file pcbi.1005659.s002.zip › OpenMM-7.0.1-Source/docs/api-python/generated/simtk.openmm.app.desmonddmsfile.DesmondDMSFile.html]

DesmondDMSFile — OpenMM 7.0.1.dev-5e86c4f documentation


# DesmondDMSFile¶

*class* `simtk.openmm.app.desmonddmsfile.``DesmondDMSFile`(*file*)¶
:   DesmondDMSFile parses a Desmond DMS (desmond molecular system) and
    constructs a topology and (optionally) an OpenMM System from it

    `__init__`(*file*)¶
    :   Load a DMS file

        |  |  |
        | --- | --- |
        | Parameters: | **file** (*string*) – the name of the file to load |

    Methods

    |  |  |
    | --- | --- |
    | `__init__`(file) | Load a DMS file |
    | `close`() | Close the SQL connection |
    | `createSystem`([nonbondedMethod, ...]) | Construct an OpenMM System representing the topology described by this |
    | `getPositions`() | Get the positions of each atom in the system |
    | `getProvenance`() | Get the provenance string of this system |
    | `getTopology`() | Get the topology of the system |

    `getPositions`()¶
    :   Get the positions of each atom in the system

    `getTopology`()¶
    :   Get the topology of the system

    `getProvenance`()¶
    :   Get the provenance string of this system

    `createSystem`(*nonbondedMethod=NoCutoff*, *nonbondedCutoff=Quantity(value=1.0*, *unit=nanometer)*, *ewaldErrorTolerance=0.0005*, *removeCMMotion=True*, *hydrogenMass=None*)¶
    :   Construct an OpenMM System representing the topology described by this
        DMS file

        |  |  |
        | --- | --- |
        | Parameters: | - **nonbondedMethod** (*object=NoCutoff*) – The method to use for nonbonded interactions. Allowed values are   NoCutoff, CutoffNonPeriodic, CutoffPeriodic, Ewald, or PME. - **nonbondedCutoff** (*distance=1\*nanometer*) – The cutoff distance to use for nonbonded interactions - **ewaldErrorTolerance** (*float=0.0005*) – The error tolerance to use if nonbondedMethod is Ewald or PME. - **removeCMMotion** (*boolean=True*) – If true, a CMMotionRemover will be added to the System - **hydrogenMass** (*mass=None*) – The mass to use for hydrogen atoms bound to heavy atoms. Any mass   added to a hydrogen is subtracted from the heavy atom to keep their   total mass the same. |

    `__delattr__`¶
    :   x.\_\_delattr\_\_(‘name’) <==> del x.name

    `__format__`()¶
    :   default object formatter

    `__getattribute__`¶
    :   x.\_\_getattribute\_\_(‘name’) <==> x.name

    `__hash__`¶

    `__reduce__`()¶
    :   helper for pickle

    `__reduce_ex__`()¶
    :   helper for pickle

    `__repr__`¶

    `__setattr__`¶
    :   x.\_\_setattr\_\_(‘name’, value) <==> x.name = value

    `__sizeof__`() → int¶
    :   size of object in memory, in bytes

    `__str__`¶

    `close`()¶
    :   Close the SQL connection

High performance molecular simulation on GPUs


### Navigation

- Application Layer
  - Loaders and Setup
  - Representation and Manipulation
  - Simulation
  - Reporting Output
  - Extras
- Library Layer

©2015, Stanford University and the Authors.
|
Powered by Sphinx 1.3.1
& Alabaster 0.7.7
|
Page source
